# Chronic Gestational Stress Leads to Depressive-Like Behavior and Compromises Medial Prefrontal Cortex Structure and Function during the Postpartum Period

**DOI:** 10.1371/journal.pone.0089912

**Published:** 2014-03-03

**Authors:** Benedetta Leuner, Peter J. Fredericks, Connor Nealer, Christopher Albin-Brooks

**Affiliations:** Departments of Psychology and Neuroscience, The Ohio State University, Columbus, Ohio, United States of America; University of Regensburg, Germany

## Abstract

Postpartum depression, which affects approximately 15% of new mothers, is associated with impaired mother-infant interactions and deficits in cognitive function. Exposure to stress during pregnancy is a major risk factor for postpartum depression. However, little is known about the neural consequences of gestational stress. The medial prefrontal cortex (mPFC) is a brain region that has been linked to stress, cognition, maternal care, and mood disorders including postpartum depression. Here we examined the effects of chronic gestational stress on mPFC function and whether these effects might be linked to structural modifications in the mPFC. We found that in postpartum rats, chronic gestational stress resulted in maternal care deficits, increased depressive-like behavior, and impaired performance on an attentional set shifting task that relies on the mPFC. Furthermore, exposure to chronic stress during pregnancy reduced dendritic spine density on mPFC pyramidal neurons and altered spine morphology. Taken together, these findings suggest that pregnancy stress may contribute to postpartum mental illness and its associated symptoms by compromising structural plasticity in the mPFC.

## Introduction

Depression is the most common complication following childbirth experienced by an estimated 15–20% of women [1], [2]. The symptoms of postpartum depression (PPD) mimic those of major depression and can include anhedonia, disrupted sleep and appetite, persistent feelings of sadness and hopelessness as well as cognitive impairment [3], [4]. In addition, considerable evidence indicates that PPD is accompanied by inadequate caregiving which is characterized by low levels of physical contact, infrequent feeding behaviors, and attachment issues [3], [5]. As a result, the children of depressed mothers tend to have abnormal physical, social, and cognitive development and are themselves more likely to experience psychopathology later in life [6], [7]. Thus, PPD not only negatively impacts the mother's well-being but also has devastating consequences for the child.

PPD is thought to arise at least in part because of the drastic fluctuations in hormones that a woman's body undergoes after birth [8]. Besides hormonal changes, numerous other variables are known to enhance vulnerability to PPD. One of the strongest predictors for the emergence of PPD is exposure to chronic stress during pregnancy [1], [9]. Similarly, animal studies have demonstrated that chronic stress during pregnancy can increase depressive-like behavior during the postpartum period and impair maternal care [10]–[12]. Moreover, in rodents, some postpartum-related adaptations such as attenuated anxiety [13] and improved spatial ability [14] are abolished by gestational stress. However, little is known about the impact of pregnancy stress on the neural substrates regulating emotional behavior, maternal care, and cognitive function during the postpartum period.

The medial prefrontal cortex (mPFC) is a brain region that has been linked to stress, cognition, maternal care, and mood disorders including PPD [15]–[20]. In the mPFC, neurons undergo experience-dependent structural reorganization including changes in the size, shape, and number of dendritic spines, sites of excitatory synapses [21]. These spine alterations in the mPFC may be the basis of changes in behavior [20], [22]. For example, during the postpartum period, mPFC spine density is increased and this coincides with improved behavioral flexibility on an attentional set shifting task that relies on the mPFC [23]. In contrast, aberrant spine changes in the mPFC have been observed following stress [20], [24]–[27] and have been associated with stress-related disorders including depression [28]. Taken together, these data suggest that some of the behavioral features associated with PPD may involve stress-induced spine modifications in the mPFC. To investigate this possibility, we examined the effects of chronic gestational stress on emotional behavior and maternal care as well as mPFC structure and cognitive function. We found that chronic gestational stress resulted in postpartum maternal care deficits, increased depressive-like behavior, and impaired performance on an attentional set shifting task that relies on the mPFC. Furthermore, exposure to chronic stress during pregnancy reduced dendritic spine density on mPFC pyramidal neurons and altered spine morphology. Overall, these findings suggest that pregnancy stress may contribute to postpartum mental illness and its associated symptoms by compromising structural plasticity in the mPFC.

## Materials and Methods

### Ethics statement

All experimental procedures were approved by The Ohio State University Institutional Animal Care and Use Committee (Protocol No. 2011A00000005) and conformed to the U.S. National Institutes Health Guide for the Care and Use of Laboratory Animals.

### Animals

Timed pregnant female Sprague-Dawley rats were purchased from Taconic (Germantown, NY). Upon arrival on gestation day 4 (GD4), rats were housed individually on a 12 h light/dark cycle and provided food and water *ad libitum*. The day of birth was designated as postpartum day 0 (PD0) and litters were culled to 10 pups (4–6 males, 4–6 females) on PD1.

### Chronic stress procedure

From GD7-13, pregnant rats were subjected to 20 min of inescapable swim stress twice daily by being placed into a cylindrical container (37×30 cm) of room temperature water (25±0.5°C). From GD14-20, pregnant rats were placed into Plexiglas restrainers twice daily for 30 min of restraint stress. For the forced swim test (FST) experiment, the stress paradigm was modified such that rats were exposed to 30 min of restraint stress twice daily from GD7-20 in order to avoid any possible effects of prior exposure to swim stress on behavior in the FST. On each day, the stressors were administered at least 4 h apart during the light phase. Unstressed females were left undisturbed.

### Validation of the stress paradigm, maternal care, and anxiety like-behavior

Beginning on the day of stress exposure, rats (no stress, n = 9; stress, n = 10) were weighed daily until the time of sacrifice. To assess litter characteristics, pups were weighed, counted, and sexed on PD1 before culling. Litters were also weighed daily until the time of sacrifice. For maternal behavior testing on PD2, postpartum females were habituated to the testing room for 30 min and then pups removed from the nest. Following a 15 min separation, pups were returned to the home cage and a 30 min maternal behavior observation was done during which dams were assessed for the percentage of time spent engaged in the following: pup licking and grooming, nest building, arched-back nursing, off the nest. 5 min following the completion of maternal behavior testing, rats underwent testing for anxiety-like behavior using the elevated plus maze (EPM). The EPM consisted of a cross-shaped platform with four arms (height: 50 cm, width: 10 cm, length: 50 cm), two of which were enclosed by walls 50 cm in height. Rats were placed in the center of the platform (10×10 cm) facing a junction between an open and closed arm and allowed to explore for 10 min. The number of entries into the open arms and the percentage of time spent in the open arms [(time in open arms/time in open and closed arms)×100] were used as measures of anxiety-like behavior. Locomotor activity independent of anxiety was assessed using the number of closed arm entries [29]. Both behavioral tests were conducted under fluorescent overhead ambient lighting (∼700 lux), digitally recorded, and later scored blind for the behaviors described by a trained observer using BEST Collection and Analysis software (Education Consulting Inc., Hobe Sound, FL). On PD21, rats were anesthetized with Euthasol and perfused with 4% paraformaldehyde in 0.1M PBS. The adrenal glands were removed, pruned of fat tissue, weighed, and relative adrenal weight calculated [total weight of left and right adrenals (mg)/body weight (g)]. Brains were also removed for immunohistochemical analysis of PSD-95 immunoreactivity (PSD-95-ir).

### PSD-95 immunofluorescence, imaging, and quantification

Forty-µm thick coronal sections were cut with a Vibratome and stored at 4°C in 0.1M PBS until histological processing. A 1∶6 series of free-floating sections containing the anterior cingulate and prelimbic regions of the mPFC were rinsed in 0.1M PB and incubated in 1% sodium borohydride for 10 min to remove residual aldehydes. Next, sections were rinsed in 0.1M TBS then blocked and permeabilized in a solution of 5% normal goat serum (NGS), 3% bovine serum albumin (BSA), and 0.3% Triton-X 100. A rabbit anti-PSD-95 antibody (1∶1000; Cell Signaling Technology, Danvers, MA) in a 0.5M TBS solution of 1% NGS, 3% BSA, and 0.3% Triton-X 100 was then applied. The PSD-95 antibody detects an expected single 95 kDa band on Western blot from rat and mouse brain tissue and preabsorption with the immunogen peptide eliminates all staining (manufacturer's technical information). The staining pattern in rat retina is identical to that seen using a monoclonal PSD-95 antibody made against a different portion of the protein (manufacturer's technical information). The discrete punctate immunofluorescent labeling is consistent with the pattern seen in the rodent brain in other studies that have employed this PSD-95 antibody [30]. Following a 24 h incubation at 4°C, sections were then rinsed and incubated for 3 h at room temperature in goat anti-rabbit Alexa Fluor 488 (1∶600; Invitrogen, Grand Island, NY) in 0.1M TBS. Sections were rinsed, mounted onto Superfrost slides, dried, and coverslipped with Prolong Gold Antifade mounting solution.

Images of PSD-95-ir were taken 200–300 µm from the pial surface using a Nikon 90i confocal microscope with a 60× objective and a 2× optical field zoom. An average of 7 image stacks were analyzed per brain. Image stacks consisted of 63.65 µm×63.65 µm through 2 µm depth (taken with a z-step of 0.2 µm) for a total volume of 8102.65 µm^3^. Each image stack was analyzed using NIS-Elements Analysis software for the number of PSD-95-ir puncta. Briefly, a background area containing little to no positive stain was used to establish a threshold which was calculated as the background intensity plus five times the standard deviation. An additional size threshold was applied to eliminate any objects with an area less than 0.01 µm^2^ as these were believed to represent pixel artifacts and not positive stain.

### Depressive-like behavior

The FST was used to assess depressive-like behavior in a separate cohort of stressed (n = 8) and unstressed (n = 8) mothers. Following parturition, rats were left undisturbed until PD21 at which time they were individually placed for 15 min into Plexiglas cylinders (49.5×30 cm) filled to depth of 30 cm with 25±0.5°C water, towel-dried, and returned to their home cage. 24 h later, rats were returned to the same apparatus for 5 min and the session digitally recorded. The percentage of time spent immobile [(time spent floating in the water only making movements necessary to maintain the head above water/total test time)×100] was later measured blind by a trained observer using BEST.

### Attentional set shifting

The attentional set shifting test was conducted on separate cohorts unstressed and stressed postpartum rats (n = 6/group) [23]. In the test, rats were trained to recover a food reward (1/3 Froot Loop) buried in terra cotta digging pots (internal diameter and depth, 10 cm) filled with digging medium and covered with various textures. The set shifting apparatus was an opaque Plexiglas box (50×40×30 cm) consisting of 3 areas - a starting/holding area (16×40×30 cm) separated by a sliding opaque divider from a testing area subdivided by a barrier into two (34×20×30 cm) compartments where the digging pots were placed.

Rats were food restricted and maintained at 85% of their baseline weight for 10 d prior to testing (which occurred on PD21-22) to ensure sufficient motivation to perform the task. On each of these days, rats were placed in the apparatus and allowed to freely explore for 30 min without the presence of pots or the divider. During the last day of acclimation, rats were placed in the apparatus with the divider in place. A pot filled with cob bedding with a reward on top was located in each section of the testing area. Rats were given 30 min to consume the rewards.

Following acclimation, rats began the first of two phases of training occurring on consecutive days. On each of these days, rats were brought to the testing room and allowed to acclimate for 30 min. *Day 1 training* began with rats being placed in the start compartment with the dividing wall in place. Two plain pots filled with cob bedding were baited and placed behind the divider, one in each section of the test area. A trial began when the divider was removed giving the rat access to both pots. Rats were initially given four trials (120 s each) to explore and dig in both pots. Following the discovery period, rats underwent training in 4 stages in which the reward was: 1) placed on top of the cob; 2) placed under a thin layer of cob; 3) buried beneath ∼2 cm of cob; 4) buried under ∼4 cm of cob. For all stages, rats were given 120 s to retrieve one reward. If rats failed to do so, the trial was repeated. Progression to the next stage required that rats retrieve one reward within 120 s for 6 consecutive trials.

During *Day 2 training*, rats were taught to differentiate between two perceptual dimensions by presenting them with pairs of pots that differed on only one dimension. These included a digging medium discrimination (shredded paper vs. shredded latex gloves) and a texture discrimination (sand paper vs. duct tape). The stimuli used in training were not used again for testing. Throughout training, the correct pot was baited with the food reward while the incorrect pot contained crushed Froot Loop in the digging medium to prohibit the animal from using the scent of the reward to guide its behavior. The left-right positioning of the baited container across trials was randomized. Prior to the start of each trial, the rat was confined within the larger holding portion of the apparatus with the divider wall in place. The trial began when the divider wall was lifted giving the animal access to the two test sections, each containing a pot. Rats were initially given four trials (120 s each) to explore and dig in both pots. Following the discovery period, the rat was given 120 s to dig in either pot. In the event of an incorrect choice, the divider wall was immediately replaced so that the animal was not allowed access to the alternate pot. If an animal did not dig within 120 s, the partition was lowered forcing the rat back into the waiting area. In either case, the trial was aborted and recorded as an error. Trials for each discrimination were continued until the animal reached a response criterion of 6 correct consecutive digs in the baited pot.

On Day 3, rats underwent *testing* on a series of 5 discriminations presented in a fixed order for all rats (Table 1). For all testing trials, rats had access to both containers, only one of which was baited with a reward. As in training, the left-right positioning of the baited container across trials was randomized and the first four trials were treated as exploration trials. For the remainder of the testing stage, the rat was given 120 s to dig in either pot. An error was recorded if the rat made an incorrect choice and dug in the unbaited pot or if the rat failed to dig. In either case, the trial was terminated and the divider wall was replaced forcing the rat back into the waiting area. Progression to the next stage of the task occurred once criterion performance (6 consecutive correct responses) was achieved. If 5 consecutive no dig trials occurred, the test was terminated and continued on the following day. Testing began with the presentation of a simple discrimination (SD) in which rats discriminated between 2 digging media in untextured containers, one of which predicted the food reward (positive stimulus). Next, in a compound discrimination (CD), a new texture dimension was introduced, but the positive stimulus was the same as in the SD. This was followed by an intradimensional attentional shift (IDS) involving two new stimuli from each stimulus dimension with digging medium remaining as the relevant dimension. The IDS was then reversed (REV), such that the formerly negative stimulus became the positive stimulus. Finally, in the extradimensional attentional shift (EDS), two new stimuli from each dimension were introduced, and the formerly task-irrelevant dimension (texture) became relevant. Stimulus pairs used are shown in Table 1.

**Table 1:** Behavioral protocol for attentional set shifting.

Discrimination Stage	Dimensions		Example Combinations	
	Relevant	Irrelevant	(+)	(−)
Simple (SD)	Medium	-	**Beads**	Gravel
Compound (CD)	Medium	Texture	**Beads**/Leather	Gravel/Denim
	Medium	Texture	**Beads**/Denim	Gravel/Leather
Intradimensional shift (IDS)	Medium	Texture	**Drierite**/Fur	Wood shavings/Reverse fur
	Medium	Texture	**Drierite**/Reverse fur	Wood shavings/Fur
Reversal (REV)	Medium	Texture	**Wood shavings**/Fur	Drierite/Reverse fur
	Medium	Texture	**Wood shavings**/Reverse fur	Drierite/Fur
Extradimensional shift (EDS)	Texture	Medium	**Velvet**/Paper towel	Reverse velvet/Hamster bedding
	Texture	Medium	**Velvet**/Hamster bedding	Reverse velvet/Paper towel

Representative example of stimulus pairs and the progression through stages of the attentional set shifting task. Digging medium was the initial discriminative stimulus dimension, shifting to texture in the EDS stage. For each stage, the positive stimulus is in bold and is paired randomly across trials with the two stimuli from the irrelevant dimension.

### DiI labeling

Separate cohorts of postpartum females subjected to pregnancy stress (n = 6) were left undisturbed until PD21-22 at which time they were anesthetized with Euthasol and perfused with 1.5% paraformaldehyde in 25 mM PBS along with postpartum females that did not undergo pregnancy stress (n = 6). Brains were removed, postfixed for 1 h, and 200 µm thick coronal sections cut with a Vibratome. Sections were stored at 4°C in 25 mM PBS until fluorescently labeled with DiI [30]–[32]. A modified gene gun (Helios Gene Gun System; BioRad, Hercules, CA) powered by helium gas (100 psi) was used for ballistic delivery of tungsten particles (1.3 µm diameter, BioRad) coated with the carbocyanine fluorescent dye DiI (Invitrogen) onto tissue sections. Labeled tissue was stored in 25 mM PBS overnight at room temperature to allow for DiI diffusion through cellular membranes. Sections were then rinsed, mounted onto Superfrost slides (Fisher Scientific, Pittsburgh, PA), dried, and coverslipped with Prolong Gold Antifade mounting solution (Invitrogen).

### Confocal imaging for quantification of spine density and spine morphology

A Nikon 90i confocal microscope was used to image dendritic segments with a 100× oil immersion objective by capturing image stacks at 0.2 µm interval steps in the z-plane. Fluorescence emitted by DiI was visualized by excitation with an argon laser (568 nm). Spine density measurements were conducted on confocal image stacks using Nikon Elements software. DiI labeled neurons in layer 2/3 of the anterior cingulate and prelimbic regions of the mPFC were analyzed. For every cell, dendritic segments selected for analysis were: (1) each 10–25 µm long; (2) on secondary or tertiary dendrites; (3) ≥75 µm away from the soma for apical dendrites and 50 µm for basal dendrites. Five randomly selected dendrites satisfying these criteria on four to five cells per animal were examined. Spine densities were calculated by dividing the total number of spines by the length of the dendritic segment and the data expressed as the number of spines/10 µm.

For spine morphology, Z-stacks were deconvolved using AutoQuantX (MediaCybernetics, Rockville, MD) and morphological analysis performed using NeuronStudio software [33]. Spines were classified as thin or mushroom if the ratio of their maximum head diameter to maximum neck diameter was >1.1. Of the spines that met this criterion, those with a maximum head diameter <0.4 µm were classified as thin spines and those with maximum spine head diameters >0.4 µm were classified as mushroom spines. Spines with a head∶neck ratio <1.1 were classified as stubby spines. An average of 5 dendrites per neuron on five neurons per animal totaling ∼9,300 apical spines and ∼8,400 basal spines per experimental group were analyzed. For each animal, the ratio of spines in each morphological category was calculated. To minimize bias, all analyses were done blind to the experimental condition.

### Statistical Analysis

Group data are reported as the mean ± SEM. Since we had *a priori* hypothesized that gestational stress would have detrimental effects on maternal and pup parameters as well as FST, EPM, and maternal behaviors, these data were analyzed using one-tailed, unpaired Student's t-tests. Trials to reach criterion during the attentional set shifting task was analyzed using two-way repeated measures ANOVA with task phase as a within subjects factor and group (no stress or stress) as a between subjects factor. Spine density (apical and basal) and PSD-95-ir were analyzed using two-tailed, unpaired Student's t-tests while spine morphology was analyzed using two-way ANOVA with group (no stress or stress) and spine type (stubby, thin, or mushroom) as factors. *Post hoc* tests were performed using Newman-Keuls (one-way ANOVA) or Bonferroni (two-way ANOVA). When variances were found to be significantly different, Welch's correction was applied. All analyses were conducted using GraphPad Prism 5.0 software (La Jolla, CA) with significance set at *p*≤0.05.

## Results

### Effects of gestational stress on physiological parameters of mothers and pups

**Figure 1:**
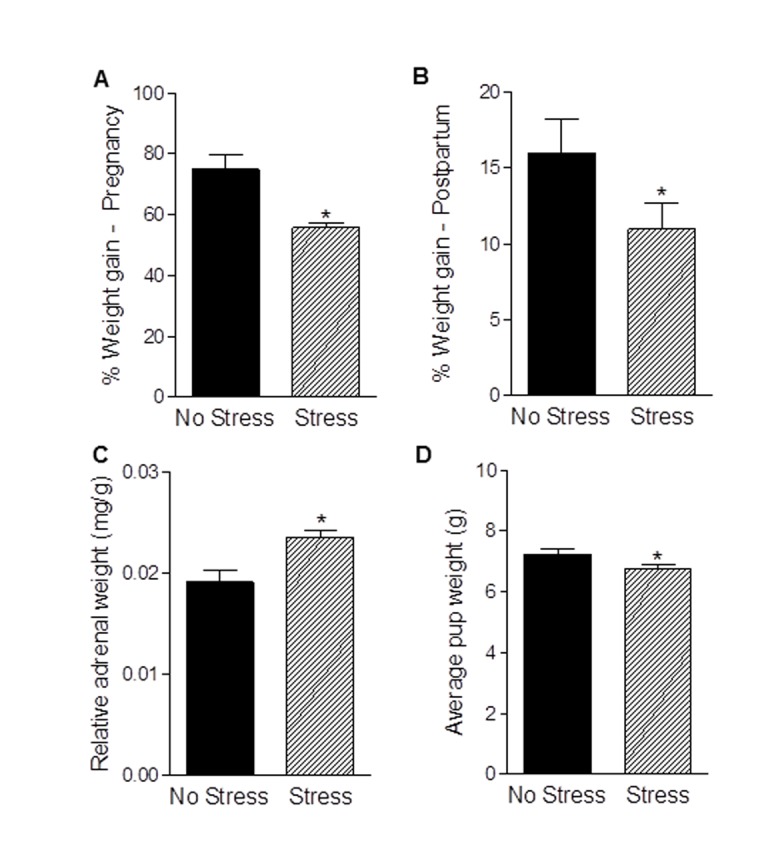
Effects of gestational stress on physiological parameters of mothers and pups. Exposure to chronic stress during pregnancy decreased gestational (a) and postpartum (b) weight gain but increased relative adrenal weight (c). Gestational stress also led to a reduction in average pup weight at birth (d). Bars represent mean ± SEM, * *p*<0.05.

Chronic stress exposure reduced body weight gain during pregnancy (t_9_ = 3.61, *p*<0.005; Fig. 1a) and the postpartum period (t_17_ = 1.79, *p*<0.05; Fig. 1b). In addition, relative adrenal weights were increased in postpartum females exposed to gestational stress as compared to postpartum females who were unstressed (t_17_ = 3.44, *p*<0.005; Fig. 1c). There was no effect of pregnancy stress on the length of pregnancy, which was approximately 22 d for both unstressed and stressed females (*p*>0.05). In addition, pregnancy stress did not affect litter size (no stress: 12.7±0.55 pups; stress: 12.4±0.99 pups; *p*>0.05), although there was a reduction in average pup weight at birth (t_17_ = 1.86, *p* = 0.04; Fig. 1d). Postnatal body weight gain of offspring born to stressed and unstressed mothers did not differ (no stress: 732.6±43.3%; stress: 741.9±43.1%; *p*>0.05).

### Gestational stress increases depressive-like behavior and has detrimental effects on maternal care

**Figure 2:**
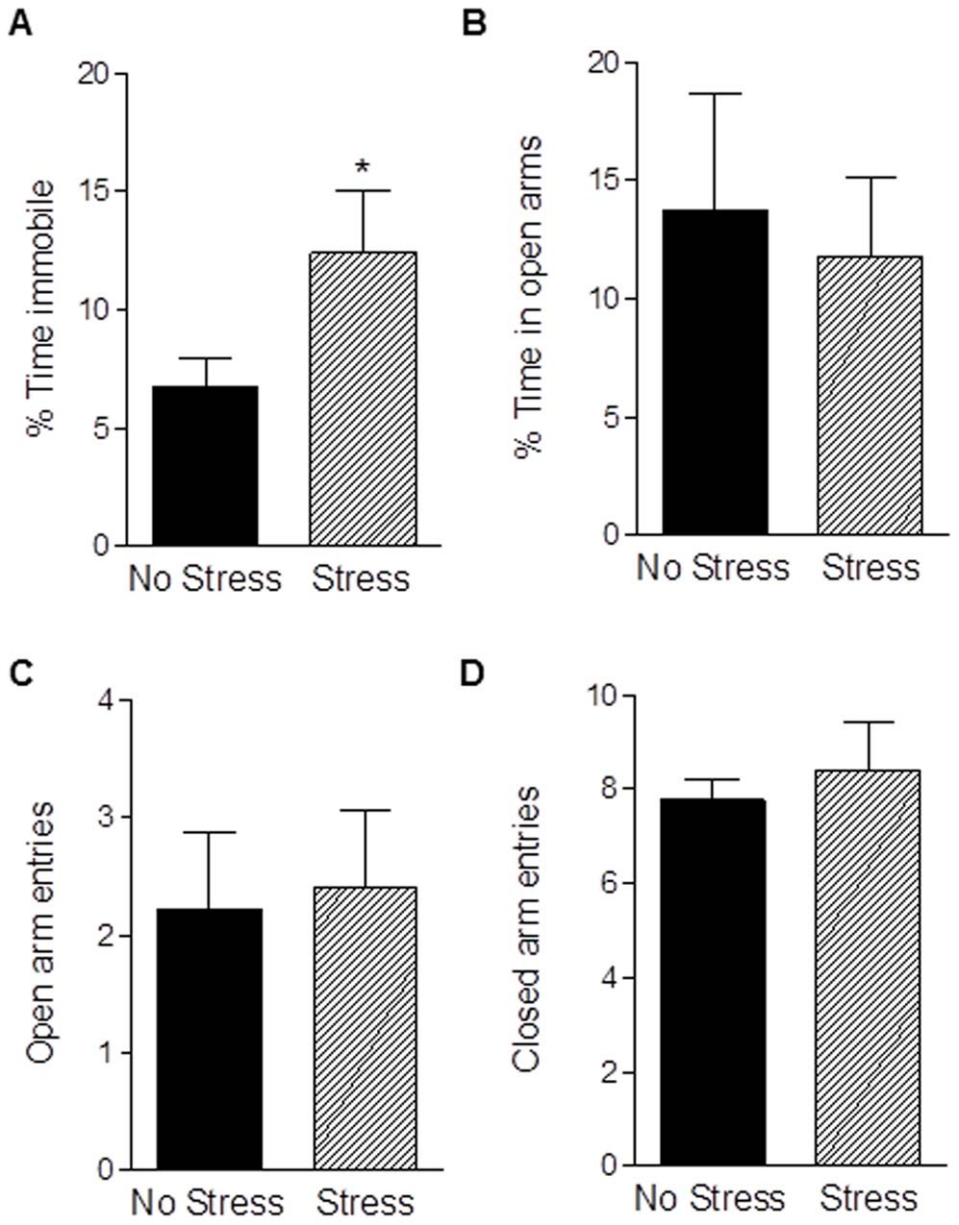
Chronic gestational stress increases postpartum depressive-like, but not anxiety-like, behaviors. Exposure to chronic stress during pregnancy increased the percentage of time postpartum females spent immobile in the forced swim test (a). Chronic gestational stress did not alter anxiety-like behavior in the elevated plus maze. Stressed and unstressed mothers spent a similar percentage of time (b) and made a similar number of entries (c) into the open arms. Locomotor activity, as assessed by closed arms entries in the EPM (d), was unaffected by gestational stress. Bars represent mean ± SEM, * *p*<0.05.

**Figure 3:**
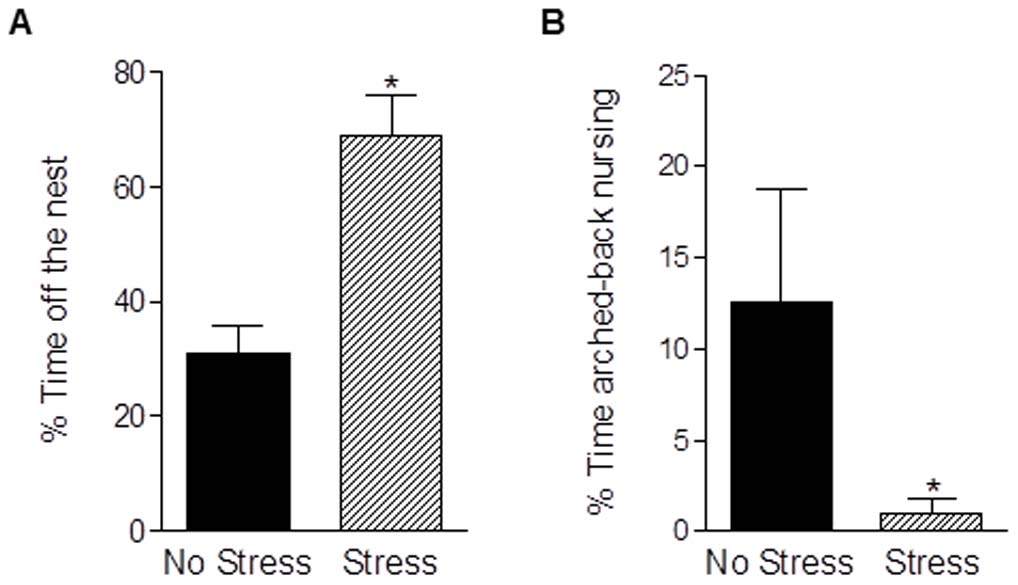
Chronic gestational stress impairs postpartum maternal care. Exposure to chronic stress during pregnancy increased the percentage of time postpartum females spent away from the nest (a) and reduced arched-back nursing (b). Bars represent mean ± SEM, * *p*<0.05.

Chronic pregnancy stress increased depressive-like behavior as demonstrated by a greater percentage of time spent immobile in the FST (t_14_ = 1.96, *p* = 0.04; Fig. 2a). There was no effect of pregnancy stress on anxiety-like behavior in the EPM - unstressed and stressed mothers spent a similar amount of time in the open arms and made a similar number of entries into the open arms (*p's*>0.0.5; Fig. 2b–d). However, exposure to gestational stress attenuated postpartum maternal care. Compared to unstressed mothers, postpartum females stressed in pregnancy spent more time off the nest away from pups (t_17_ = 4.23, *p*<0.0005; Fig. 3a) and a reduced amount of time arched-back nursing (t_17_ = 1.98, *p*<0.05; Fig. 3b). Other maternal behaviors including nest building and pup licking/grooming were unaffected (data not shown; *p's*>0.05).

### Gestational stress impairs reversal learning and extradimensional set shifting

**Figure 4:**
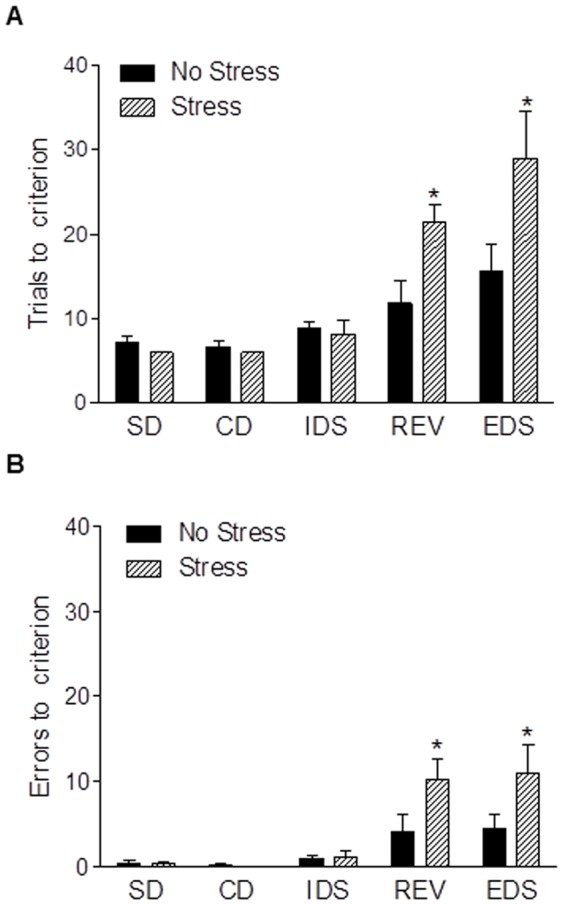
Chronic gestational stress impairs cognitive flexibility. Compared to postpartum females who were unstressed, postpartum females stressed in pregnancy showed impairments on the reversal (REV) and extra dimensional (EDS) phases of the attentional set shifting task as demonstrated by more trials (a) and errors (b) to reach criterion. The number of trials (a) and errors (b) to reach criterion for the simple discrimination (SD), compound discrimination (CD) and intradimensional shift (IDS) did not differ between unstressed and stressed mothers. Bars represent mean ± SEM, * *p*<0.05.

Fig. 4 shows the effects of chronic gestational stress on the trials to reach criterion and errors to reach criterion for each phase of the attentional set shifting task. For trials to criterion (Fig. 4a), two-way repeated measures ANOVA revealed main effects of group (F_1,10_ = 8.63, *p*<0.05) and task phase (F_4,40_ = 17.27, *p*<0.0001) and a task phase by group interaction (F_4,40_ = 4.04, *p*<0.01). Post hoc analysis showed that postpartum females stressed in pregnancy required more trials to reach criterion on the REV (*p*<0.05) and EDS phases (*p*<0.01) relative to unstressed postpartum females. For errors to criteria (Fig. 4b), there was a near significant main effect of group (F_1,10_ = 3.73, *p* = 0.08), a main effect of task phase (F_4,40_ = 14.07, *p*<0.0001), and a task phase by group interaction (F_4,40_ = 2.93, *p*<0.05) with post hoc analysis revealing that more errors to reach criterion were made on the REV and EDS phases by postpartum females stressed in pregnancy (*p's*<0.05). There were no significant differences between stressed and unstressed postpartum females in trials to criteria or errors to criteria on any other task phase (*p's*>0.05).

### Gestational stress reduces mPFC dendritic spine density and alters spine morphology

**Figure 5:**
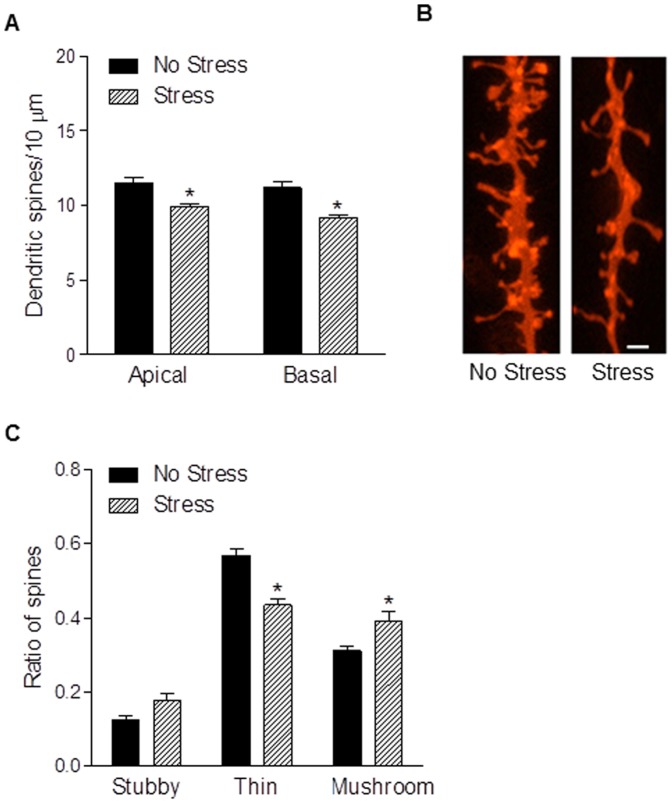
Chronic gestational reduces mPFC dendritic spine density and alters spine morphology. Postpartum females subjected to chronic gestational stress had fewer dendritic spines on apical and basal dendrites of layer 2/3 pyramidal neurons in the mPFC (a). Representative DiI labeled dendritic segments, scale bar = 1 µm (b). Exposure to chronic gestational stress also caused a shift in spine morphology such that there was a decrease in the proportion of thin spines but an increase in the proportion of mushroom spines in stressed mothers (c). Bars represent mean ± SEM, * *p*<0.05.

**Figure 6:**
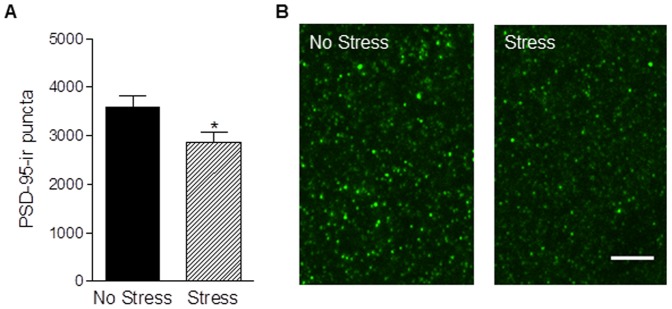
Chronic gestational reduces the number of PSD-95-ir puncta in the mPFC. Postpartum females stressed in pregnancy had fewer PSD-95-ir puncta in the mPFC (a). Representative confocal images of PSD-95-ir puncta in the mPFC, scale bar = 5 µm (b). Bars represent mean ± SEM, * *p*<0.05.

Compared to postpartum females who were unstressed, postpartum females exposed to chronic gestational stress had 13% fewer spines on apical (t_11_ = 3.75, *p*<0.005) and 18% fewer spines on basal (t_11_ = 4.04, *p*<0.005) dendrites of layer 2/3 pyramidal cells in the mPFC (Fig. 5a). In order to corroborate dendritic spine density results with an excitatory postsynaptic marker, we quantified PSD-95-ir puncta in mPFC layer 2/3 with confocal microscopy and found that the density of PDS-95-ir puncta was reduced by approximately 20% in stressed mothers (t_17_ = 2.41, *p*<0.05; Fig. 6).

To address whether the stress-induced changes in mPFC spine density reflect a non-specific regulation of all types of dendritic spines or specific subtypes, we classified spines into three well described morphologic sub-types: thin, mushroom, and stubby. We found that chronic pregnancy stress caused a shift in spine morphology on apical dendrites of mPFC layer 2/3 pyramidal neurons (Fig. 5b). Two-way ANOVA revealed no main effect of group (F_1,33_ = 0.004, *p* = 0.95), a main effect of spine type (F_2,33_ = 198.2, p<0.0001), and group by spine type interaction (F_2,33_ = 21.45, *p*<0.001). Post hoc analysis showed that gestational stress led to a significant decrease in the proportion of thin spines (*p*<0.001) but an increase in the proportion of mushroom spines (*p*<0.05). Gestational stress did not affect the ratio of stubby spines (*p*>0.05) or spine morphologies on basal dendrites of mPFC pyramidal neurons (F_2,33_ = 0.58, *p*>0.05).

## Discussion

Here we show that postpartum rats exposed to chronic stress in pregnancy exhibit depressive-like behavior that is accompanied by physical and behavioral changes reflecting some of the symptoms seen in depressed mothers including reduced body weight gain and impaired maternal care. In addition, our data demonstrate that chronic gestational stress impairs mPFC cognitive function while decreasing mPFC spine density and altering spine morphology. Overall, these data indicate that chronic gestational stress may be a potentially useful model to study the neural mechanisms underlying PPD and its associated maternal and cognitive deficits which our results suggest may involve compromised structural plasticity in the mPFC.

In this study, mothers who were exposed to chronic stress during pregnancy displayed several classical stress-related changes including reduced body weight gain as well as enlarged adrenals. These results are in line with previous work in postpartum females using different stress protocols [13], [34] and confirm the validity of our stress procedure. Also consistent with prior observations [10], [11] is the demonstration that postpartum females subjected to chronic stress during pregnancy exhibit higher immobility in the FST, suggestive of depressive-like behavior. However, while others have found increased postpartum anxiety following gestational stress [13], [34] we did not detect any changes in anxiety-like behavior. The reason for this discrepancy may be related to the type and/or duration of the stressor used or specifics of the testing procedures (i.e. handling, acclimation to the testing room, lighting conditions, duration and time of testing) all of which are known to impact behavior in the EPM [29], [35].

It is widely known that depression in mothers can disrupt parenting behaviors. Among the deficits often seen in depressed mothers are low levels of physical interaction and infrequent feedings behaviors [3], [5]. Similarly, we found maternal care deficits in postpartum females subjected to chronic stress during pregnancy which were characterized by a reduction in the amount of time spent arched-back nursing and more time spent off the nest away from pups. These data suggest that maternal care is adversely affected by pregnancy stress in ways that can accompany PPD. They also add to existing rodent studies reporting stress-induced deficits in maternal care and a negative association between depressive-like behavior and maternal care [10]–[12], [36]–[38]. However, even though stressed mothers spent less time on the nest and less time nursing, body weights of offspring from stressed mothers did not differ from those of unstressed mothers beyond PD1. This raises the possibility that the maternal care deficits observed on PD2 did not persist but this seems unlikely as prior work has shown impaired maternal care resulting from gestational stress over postpartum days 1–10 [10]. Instead, the amount of time the offspring of stressed mothers spent nursing may have been sufficient for feeding or perhaps although they nursed less, the nursing bouts were more intense resulting in adequate milk consumption.

In addition to altering emotional and maternal behaviors, chronic gestational stress negatively impacted cognitive function. We have previously shown that compared to virgin females without reproductive or maternal experience, postpartum females exhibit improved performance on the EDS phase of the attentional set shifting task [23]. Here we extend this work and show for the first time that gestational stress impairs extradimensional set shifting as well as reversal learning, measures of behavioral flexibility [39]. Cognitive flexibility is impaired by stress in rodents and humans and these impairments have been suggested to be important in depression [40]–[43]. Deficits in cognitive flexibility are also likely to have further implications during the postpartum period. Indeed, the postpartum period is a time when the survival and well-being of the offspring critically depends on the mother's ability to attend to her infant's needs which in turn requires that she be able to easily shift her attention depending on situational demands and adapt her behaviors accordingly [44], [45]. By interfering with these functions, gestational stress may thus compromise the mother's capacity to adequately care for her young. Consistent with this, mother rats who perform better on other types of attentional tasks are better mothers overall - they are less easily distracted, more attentive to their litter, and lick their pups more [46]. Similarly, in human mothers, reduced maternal sensitivity and difficulties during mother-infant interactions are positively correlated with attentional deficits [44], [45], [47]. Thus, the cognitive functions tested here may provide an important foundation for maternal behavior [48], both of which are compromised in PPD [3]–[5].

The mPFC was the focus of our morphological analysis because it has been linked to stress [20], [49], cognitive flexibility [20], [50], maternal care [15], [16], [18] and mood disorders [28], including PPD [17], [19]. Moreover, recent evidence suggests that mood disorders like depression may involve dendritic spine loss and other synaptic alterations within the PFC [28]. Dendritic spines are sites of excitatory synapses and as such reduced mPFC dendritic spine density in postpartum females subjected to chronic gestational stress likely reflects a decrease in excitatory synaptic input into the mPFC [21]. This possibility is supported by a concomitant decrease in the number of immunoreactive puncta for PSD-95, a scaffolding protein expressed specifically in the post-synaptic density of excitatory synapses [51]. A decrease in mPFC dendritic spine density has also been observed in male rats following exposure to chronic stress however this effect was evident one day after stressor cessation and was at least partially reversible following a three week recovery period [33]. In contrast, mPFC dendritic spine density was reduced in postpartum females subjected to chronic stress during pregnancy three weeks after stressor termination. This enduring effect of gestational stress on mPFC structure suggests that pregnant females may be particularly vulnerable to stress.

Because dendritic spines regulate synaptic function and plasticity and patterns of connectivity in neuronal circuits, alterations in mPFC spine density following gestational stress may be an anatomical substrate for stress-induced changes in mPFC function during the postpartum period. Stress-induced modifications in spine morphology may also have functional relevance. Depending on their shape, spines can be categorized as mushroom, thin or stubby [21]. These morphological distinctions are important because they correlate with spine motility and synaptic strength. In general, small thin spines are highly motile and plastic, and form weak synapses that are NMDA receptor dominated, whereas large mushroom spines are highly stable and form strong synapses that are AMPA receptor dominated [52]. Based on these differences, it has been proposed that thin spines are important for learning new information whereas mushroom spines mediate stable circuits linked to memory [53]. Consequently, the loss of thin spines, coupled with the increase in the proportion of mushroom spines, may render the mPFC of stressed mothers less plastic thus interfering with their ability to demonstrate cognitive flexibility. An analogous loss of thin spines, which correlated with impaired cognitive flexibility in a PFC-mediated task, has been demonstrated in the PFC of the aged rhesus monkey [54].

While extra-dimensional set shifting relies on the mPFC, reversal learning is supported by the orbitofrontal cortex (OFC) [50], [55]. Thus, the spine modifications we observed in the mPFC likely relate to the EDS, but not REV, deficits in stressed mothers. Compared to the mPFC, little is known regarding dendritic spine plasticity in the OFC and the behavioral correlates of those changes. Although a link between OFC spines and reversal learning hasn't been established, a recent study has shown anhedonia and dendritic spine elimination in the OFC as a result of chronic stress hormone exposure suggesting that OFC spine loss may confer vulnerability to depressive symptomatology [56]. The effects of gestational stress on dendritic spine plasticity in the postpartum OFC and its relationship to impaired reversal learning and depressive-like behavior warrant further investigation. In addition, dendritic spines in limbic regions such as the hippocampus, amygdala and nucleus accumbens are all sensitive to stress and/or glucocorticoids [22], [27], [56], [57]. But other than one recent report demonstrating increased mushroom spine density on hippocampal CA3 pyramidal neurons following postpartum corticosterone administration [58], little is known about how dendritic spines in these brain regions of postpartum females respond to stress and their possible contribution to stress-related modifications in depressive, caregiving, and cognitive behaviors.

The mechanisms underlying the adverse effects of chronic gestational stress on mPFC structure and function have yet to be elucidated but there are several possible candidates. For example, the mPFC expresses receptors for glucocorticoids making it a target for stress hormone actions [49] and work in human mothers supports a link between elevated cortisol, impaired cognitive function and lower maternal sensitivity [48]. However, while administration of high levels of glucocorticoids during pregnancy in rodents decreases body weight and impairs maternal care, it does not increase depressive-like behavior during the postpartum period [38]. Nonetheless, a role for glucocorticoids in stress-induced mPFC structural changes during the postpartum period cannot be ruled out given that chronic glucocorticoids induce spine loss in the mPFC of males [56]. Another factor that could contribute to the adverse outcomes associated with gestational stress is glutamate [59]–[62]. A recent study has found that women with PPD have increased glutamate levels in the mPFC [19]. Repeated stress is also associated with increased glutamate neurotransmission in the mPFC [63] and excessive glutamate can lead to dendritic spine loss [64]. Moreover, the detrimental effects of chronic stress on depressive-like behavior and mPFC spine density are reversed by glutamate NMDA receptor antagonists [26]. Taken together, these data raise the possibility that gestational stress may have adverse effects on the postpartum mPFC through a glutamatergic mechanism. Finally, levels of brain derived neurotrophic factor (BDNF) have been reported to be lower in women with PPD [65] and thus could also contribute to the structural and behavioral symptoms of PPD [66]. Regardless of the exact mechanism, it is important to note that mother–infant interactions are reciprocal [67], [68]. Caregiving provided by the mother influences the development of the offspring, while in turn the offspring's affiliative behavior and demands determine how competent maternal care will be [67]. In addition, stimuli provided by the infant modify a number of physiologic and behavioral responses in the mother [68]. Thus, our study does not rule out the possibility that the behavioral and neural changes observed in stressed versus unstressed mothers may result from stressed pups having a differential effect on postpartum females. Future studies employing a cross-fostering design are necessary to dissociate the direct effects of prenatal stress versus the effects of the offspring on the mother.

The negative impact of gestational stress on the physiological, neural, and behavioral development of the offspring has been well documented and includes long-lasting changes in neuroendocrine function, cognitive ability, social behavior, emotionality as well as structural and synaptic modifications in the mPFC and other brain regions [20], [36], [69]–[72]. The damaging effects of maternal stress on offspring development involve a number of mechanisms, particularly elevated levels of maternal glucocorticoids, which can cross the placenta or be transmitted via maternal milk, as well as glucocorticoid-induced changes in maternal behavior [70], [71], [73], [74]. In contrast, few studies have explored the effects of gestational stress on the mother. Given that gestational stress is a major risk factor for PPD [1], [9], assessing the consequences of gestational stress on the mPFC can provide insights into the mechanisms underlying this disorder for which little is currently known. Ultimately, this information is likely to help in devising treatment strategies that may be useful for not only improving the mother's well-being but also for preventing the potentially devastating effects that this disorder can have on the child.
